# Lipid Based Therapy for Ulcerative Colitis—Modulation of Intestinal Mucus Membrane Phospholipids as a Tool to Influence Inflammation

**DOI:** 10.3390/ijms11104149

**Published:** 2010-10-25

**Authors:** Hannah Schneider, Annika Braun, Joachim Füllekrug, Wolfgang Stremmel, Robert Ehehalt

**Affiliations:** Department of Gastroenterology, University Hospital Heidelberg, INF 410, 69120 Heidelberg, Germany; E-Mails: Hannah.Schneider@med.uni-heidelberg.de (H.S.); Annika.Braun@med.uni-heidelberg.de (A.B.); Joachim.Fuellekrug@med.uni-heidelberg.de (J.F.); Wolfgang.Stremmel@med.uni-heidelberg.de (W.S.)

**Keywords:** phospholipids, phosphatidylcholine, mucosal barrier, ulcerative colitis, phospholipase A2

## Abstract

Ulcerative colitis (UC) is the result of an inappropriate colonic inflammatory response triggered by environmental and genetic factors. We have recently shown that mucus from UC patients has a decreased phosphatidylcholine (PC) content, while clinical trials revealed that therapeutic addition of PC to the colonic mucus alleviated the inflammatory activity. The mechanisms behind this are still unclear. We hypothesized that PC has at least two possible functions in the intestine: First, it establishes the surface hydrophobicity of the mucus and therefore protects the underlying tissue against intraluminal aggressors; recent experiments on surgical specimens revealed reduced surface tension and hydrophobicity in UC patients. Second, mucus phospholipids might also be integrated into the plasma membranes of enterocytes and thereby influence the signaling state of the mucosa. PC has been shown to inhibit TNF-α induced pro-inflammatory responses including: (1) assembly of plasma membrane actin; (2) activation of MAP kinases ERK and p38; and (3) activation of NF-κB and synthesis of pro-inflammatory gene products. Other phospholipids like phosphatidylethanolamine or sphingomyelin had no effect. PC also inhibited latex bead phagosome actin assembly, killing of *M. tuberculosis* in macrophages, and sphingosine-1-phosphate induced actin assembly in macrophages. Collectively, these results provide a molecular foundation that shows PC, firstly, as an anti-inflammatory, and secondly, as a surface hydrophobicity increasing compound with promising therapeutic potential in the treatment of inflammatory bowel disease.

## 1. Introduction

A potential positive impact of phosphatidylcholine (PC) on impaired medical conditions was already shown in rat about 80 years ago, when Best *et al.* demonstrated that the supplementation of a fat enriched diet with PC prevented the accumulation of triglycerides in the liver of healthy rats [1]. Since then, increasing evidence has been obtained for the importance of PC, especially in the development and treatment of various pathologic inflammatory conditions.

Quite a number of studies have concentrated on the role of PC in gastrointestinal damage and disease, such as the development of ulcers, bleeding and chronic inflammatory conditions like ulcerative colitis. However, also inflammation in other organs and tissues was shown to be influenced by PC: Exemplarily, oral pre-treatment with PC in a murine model of collagen-induced rheumatoid arthritis exhibited protective effects [2] and dietary PC ameliorated pleural inflammation in mice [3].

**Figure 1:**
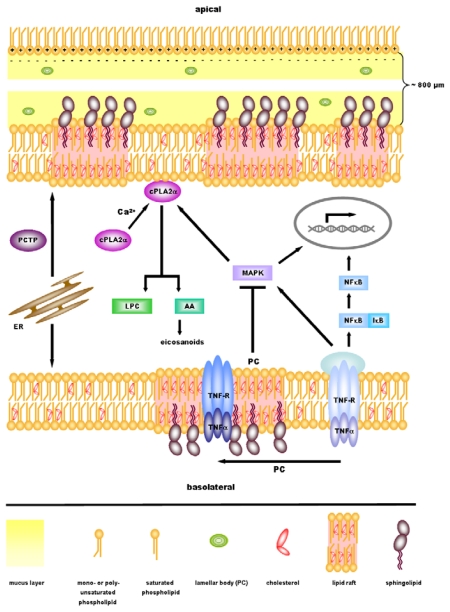
Anti-inflammatory signaling by phosphatidylcholine (PC) at the mucosal barrier. At the mucosal gastrointestinal barrier, PC is located as firstly, luminally extended monolayer and, secondly, lamellar bodies within the mucus. Interaction of the inversely charged PC headgroups and mucins establishes a hydrophobic barrier which prevents invasion of bacteria through the mucus layer. PC modulates inflammatory signaling cascades by its integration into the enterocyte membrane. It changes the compartmentation of TNF receptors to lipid rafts, inhibits NF-κB activation, prevents MAP kinase phosphorylation and subsequent activation. The cytosolic phospholipase cPLA2α is activated by calcium binding which triggers its translocation to PC enriched membranes. Degradation of PC results in the production of LPC and arachidonic acid; the latter serving as an essential precursor for eicosanoid biosynthesis. Membrane binding and catalytic activity of cPLA2α are enhanced by MAP kinase mediated phosphorylation. PC molecules are shuttled within cells from the ER to plasma membranes via PC transfer proteins (PCTP), thus allowing the modulation of the membrane PC content. The interplay of these signaling events finally results in the modulation of the inflammatory response.

Already in 1983, Lichtenberger *et al.* described a rat model of acid induced gastric bleeding and ulcer development in which the intraluminal application of a liposomal surfactant suspension acted in a positive manner [4]. From this experimental outcome emerged the hypothesis of a protective hydrophobic and PC enriched monolayer between epithelial cells and noxious luminal compounds in the gastrointestinal tract (Figure 1). This went along well with the theory of a gastric mucosal barrier as postulated by Davenport in the 1960s. Davenport hypothesized the stomach epithelium to be accessible only for lipid soluble agents as opposed to electrolytes and gastric acid (reviewed by Lichtenberger [5]). Nowadays, the concept of gastrointestinal protection has therefore been linked not only to an intact mucus and enterocyte layer, but also to the presence of PC.

By further investigating the concept of a protective gastrointestinal mucus layer, informative results on the relevance of PC could be in particular obtained during *in vitro* and *in vivo* studies on the effects of non steroidal anti-inflammatory drugs (NSAIDs) like indomethacin and diclofenac. NSAIDs display a high affinity to endogenous PC and chronic application is known to induce gastrointestinal injury such as ulceration and bleeding as a common side effect. Secretion of NSAIDs into the bile allows the interaction of the drug with bile acids, thus enhancing its damaging effect. A study of the underlying mechanisms in cell culture and model liposomes revealed that indomethacin disturbs the lipid-lipid interaction in membranes and causes membrane disruption. This effect was possibly due to the drug’s incorporation into the membranes and was enhanced by its combination with bile acids [6]. In fact, rats treated with indomethacin or diclofenac showed a reduced hydrophobicity in stomach and duodenum as well as a decrease in mucus PC [7]. The combined parenteral application of indomethacin and PC reduced the toxic side effects on the gastrointestinal system in rodents without impairing the drug’s efficacy [8]. Indomethacin treatment is therefore able to alter the structure of lipid membranes. This has two potential effects in the GI-tract: Firstly, it reduces the mucosal hydrophobicity; and secondly, it most likely interacts with enterocyte membranes, which, in turn, leads to impairment in the function of membrane proteins and downstream signaling pathways. It is tempting to speculate that the addition of PC might prevent or reverse NSAID induced intestinal damage by being incorporated into the membrane and stabilizing membrane structures. Indeed, clinical studies using a PC coupled NSAID substrate reduced GI-damage without affecting its systemic effects [9,10]. How this concept of the protective effects of PC in the GI-tract also plays a role in the pathogenesis and treatment of ulcerative colitis is discussed in this review.

## 2. Concept of Ulcerative Colitis

Ulcerative colitis (UC) belongs to the group of inflammatory bowel diseases (IBD) that represents chronic inflammatory disorders of the intestine, generally classified by histopathological and clinical features into two major entities: Crohn’s disease and ulcerative colitis. While Crohn’s disease can occur throughout the GI-tract, most commonly in the terminal ileum and colon, inflammation in UC is mainly restricted to the colon [11]. At present, the general hypothesis for the pathogenesis of inflammatory bowel disease is that it is a multifactorial, polygenetic disorder in which the immune response is triggered by luminal factors. The nature of these trigger agents is not totally clear, but, most likely, microbial agents have to be implicated [12]. Especially in the colon, where concentrations of up to one trillion microbes/mL live in a mutualistic relationship with the host, a highly effective defense barrier is necessary [13].

There is increasing evidence that a loss of tolerance to the luminal microbiota is the key in pathogenesis of IBD: Surgical diversion of the fecal stream has been shown to resolve IBD inflammation distal to the surgical side [14]. Treatment with antibiotics or probiotics has been successfully used in some cases [12]. Loss of immunological tolerance towards luminal commensals has been documented [15] and, most interestingly, in IBD the gut has lost the ability to spatially separate the bacteria from the apical membrane of the enterocytes. Normally, luminal bacteria in the gut are withheld from contact with the epithelium by an adherent mucus layer, but in UC the bacteria reside directly on the luminal surface where they probably induce and maintain the inflammatory process [16].

The loss of tolerance has traditionally been interpreted as a misbalanced mucosal adaptive immunity [17]. However, in recent years it became evident that several mechanisms that belong to the innate immunity are altered: The mucus layer is reduced in thickness [18] and has an altered mucin [19] and lipid [20,21] composition. An altered glycosylation, sulfatation, expression of MUC2 and localization of MUC1 have been documented [19,22]. Expression of antimicrobial or restitutional peptides, such as defensins [23] or trefoil factor family 3 [22], is reduced. The epithelial cell layer has an increased permeability [24] and a reduced capacity for detoxification [25]. Moreover, there are alterations in the mucus and fecal bacterial communities [26]. These data support the concept that UC is, at least in part, caused by a defect in the immediate, first line defense mechanisms that would allow bacteria to permanently invade the mucosa, thus enabling them to perpetuate and chronically change the inflammatory response. UC should therefore be classified as an “autoaggressive” rather than an autoimmunologic disease. Hence, successful strategies to treat such a disorder should not solely rely on immunosuppressive or anti-inflammatory regimens, as is the actual standard; this would target only the second line of reaction. Therapies should rather target the primary source of the disease, the impaired first line defense mechanisms. One such tool would be to reinforce the mucus layer by PC.

As mentioned above, the first evidence that PC is indeed important for the surface integrity of the GI-tract came from experiments on stomach, where the pathogenesis and treatment of gastroduodenal ulceration were the focus of research (for review see [5] and [8]). However, the concept of a protective role of intestinal PC was transferred to the lower GI-tract and a protective effect of PC could be documented in several animal models for intestinal inflammation. PC has been shown to alleviate the inflammation in acetic acid [27,28], trinitrobenzene sulfonic acid (TNBS) [29,30], NSAID [31] or dextran sulfate sodium (DSS) induced ileitis and colitis models [32].

Remarkably, animal studies revealed beneficial effects on gastrointestinal damage not only for PC but also for selected lysophospholipids. In rats suffering from TNBS induced colitis, rectal application of lysophosphatidic acid (LPA) and lysophosphatidylethanolamine (LPE) caused a decreased mucosal damage indicated by a reduction in ulcer size and depth [33]. LPE is derived from phosphatidylethanolamine, whereas LPA can be synthesized via different routes, among others from lysophosphatidylcholine (LPC). However, in a cell culture model of inflammation, anti-inflammatory effects could only be shown for PC and LPC but neither for phosphatidylethanolamine nor for sphingomyelin [34–36]. It can therefore be speculated, that different lipid mediators exhibit highly specific functions within the pro-inflammatory signaling cascade, thus making a tight regulation of lipid metabolism necessary for maintaining homeostasis.

## 3. Role of PC in Ulcerative Colitis

The first evidence that PC might play a role in the pathogenesis of human UC came from lipid analysis of mucus gained by gentle scraping of the rectal wall with a cotton wool sponge during rectoscopy. Patients suffering from ulcerative colitis in clinical remission, but not from Crohn’s disease, showed significantly reduced PC/mg dry weight compared to healthy controls [20]. These results were confirmed in patients with active disease [37] as well as by analysis of ileal and colonic mucus gained during colonoscopy and compared to total protein levels of the acquired mucus aliquots [21]. Based on these findings, clinical studies were initiated to investigate whether supplementation of the missing mucus PC would alleviate the inflammation in UC. Because rectal application by a klysma was unsuccessful (patients could not keep the lipid emulsion), PC was encapsulated with the polymer Eudragit S to allow a pH dependent release in the terminal ileum, as previously used by pharmaceutical companies for a late release of mesalazine [38]. This was necessary because, if taken orally, most supplemented PC is absorbed in jejunum. Using Eudragit S encapsulated soy lecithin allowed to increase the rectal amount several times above concentrations found in otherwise healthy subjects [37]. Proof of principle (Phase IIa/b) studies were conducted by Stremmel *et al.* [37,39,40] and showed a tremendous response. Clinical activity could be reduced in up to 90% of patients. Remission could be achieved in up to 50% after a treatment period of three months. A phase III study is planned.

Another hint that PC metabolism plays a role in pathogenesis, is the link between UC and primary sclerosing cholangitis (PSC). The occurrence of PSC is rare and its causes are unknown. However, PSC is an immunologic disorder and its appearance is often associated with ulcerative colitis. Inflammation of the bile ducts results in impaired bile flow from the liver to the duodenum. Interestingly, in a colon carcinoma derived cell line, the application of conjugated primary bile salts (CPBS) reduced endotoxin permeability through differentiated confluent cells, and decreased the production of pro-inflammatory mediators, such as TNF-α, interleukin 6 (IL-6), interleukin 8 (IL-8) and interleukin 10 (IL-10). Additional treatment with PC further reduced interleukin levels and especially diminished levels of TNF-α [41], thus indicating a synergistic anti-inflammatory action of PC and CPBS. Dysfunction in PC metabolism might therefore also play a role in PSC development.

These findings highlight the relevance of specific phospholipids, especially PC, in gastrointestinal inflammation and indicate that PC is one of the important players in the process of chronic intestinal inflammation.

## 4. Anti-Inflammatory Signaling by PC

Although the anti-inflammatory properties of PC have been shown in cell culture as well as in animal models and humans, the molecular background behind these effects is insufficiently understood. However, we hypothesized that in the gut it has two possible functions: 1. It is essential for the surface hydrophobicity of the mucus and therefore protects the underlying tissue against intraluminal aggressors. 2. Mucus phospholipids might also be integrated into the plasma membranes of enterocytes and modulate the signaling state of the mucosa [34]. The groups of Lenard M. Lichtenberger and Brian A. Hills have shown in several publications that surface hydrophobicity throughout the GI tract depends on mucus phospholipids [5]. They hypothesized that the positively charged polar headgroup of PC electrostatically interacts with the strongly negatively charged mucins, forming a monolayer with the fatty acid chains extending luminally (Figure 1). This would form a hydrophobic film that expels luminal toxins and bacteria. Hydrophobicity and surface PC concentration could indeed be linked in several publications [5,42,43]. Most interestingly, recent experiments on surgical specimens revealed reduced surface tension in UC patients in parallel to a decreased PC content [44], which indicates a probable role of this mechanism in pathogenesis of UC. However, recent findings further suggest a complex interplay of PC with various mucosal signaling pathways, including TNF-α signaling, activation of NF-κB, cytokine expression and the mitogen-activated protein kinase (MAPK) pathway, as will be discussed in the following.

*In vitro* experiments with latex bead phagosomes, which are internalized by macrophages, allow the analysis of phagosome actin assembly. This process is associated with the killing of pathogens, such as mycobacteria, that use macrophage phagosomes for intracellular survival within their respective host. Actin nucleation was found to be either activated or inhibited after application of various lipids, mostly in an ATP dependent manner. The experimental outcome suggested the modulation of the involved signaling pathways due to the integration of lipids into the phagosomal membrane [35]. Under any conditions, PC displayed inhibitory properties, thus promoting the survival of mycobacteria in macrophages [34,35]. At first sight, these results seem to indicate a role for PC in promoting the development of a pathologic condition rather than acting in a protective manner. However, this effect was due to the blocking of actin assembly, a pro-inflammatory process, which reveals rather anti-inflammatory properties of PC application.

In polarized CaCo2 cells, application of PC either from the apical or basolateral site inhibited the phosphorylation and activation of the MAP kinases p38, ERK1 and ERK2. Equally, anti-inflammatory effects of PC as well as LPC could be proofed by the downregulation of pro-inflammatory cytokines such as IL-8, monocyte chemotactic protein-1 (MCP-1) and interferon γ-inducible protein-10 (PI-10) in CaCo2 cells [34]. Furthermore, the dietary supplementation of PC in rats with collagen-induced arthritis diminished the activation of leukocytes [2]. It can be speculated, that exogenous or mucus PC is integrated into cellular membranes and regulates downstream signaling cascades by altering membrane PC content and vertical or lateral distribution within the membrane lipid bilayer. Effects of LPC might at least partially be mediated via its conversion to PC [34].

PC molecules can be transported within cells by the phosphatidylcholine transfer protein (STARD2/PCTP) which has also been speculated to have regulatory functions [45]. PCTPs bind their substrates via the steroidogenic acute regulatory protein (StAR)-related lipid transfer (START) domain, which can be found in 15 different human proteins that are involved in the control of lipid metabolism. PCTP acts as shuttle from the endoplasmic reticulum to plasma membranes in order to provide PC for restocking. STARD10, another member of the STARD2/PCTP family, equally binds PC and is assumed to export lipids into the bile. Dysfunction of START proteins has been associated with different kinds of disease, also including autoimmune disorders. However, mice lacking PCTP do not show a clear phenotype which might be due to a compensatory function of STARD10 [46].

Further potential candidates to be involved in PC signaling are members of the peroxisome proliferator activated receptor (PPAR) family, ligand activated nuclear receptors that are involved in inflammatory processes and lipid metabolism. Exemplarily, PPARγ2 has been shown to prevent lipotoxic effects by enhancing the lipid buffering capacity in organs and regulating the expansion of adipose tissue [47]. Such a protective role of PPAR family members could be shown not only for PPARγ2 but also for PPARα. Drug induced activation of this receptor is already used in order to treat human disorders of the lipid metabolism, although its endogenous ligand has not yet been identified. However, recent findings suggest 1-palmitoyl-2-oleoyl-*sn*-glycerol-3-phosphocholine to be such an endogenous PPARα ligand [48], thus hinting at the involvement of PC species in PPAR activation. It is noteworthy, that activators of PPARα had beneficial effects on altered TNF-α levels in patients with dyslipidemia [49], which might suggest a new therapeutic target for anti-inflammatory treatment by PC application.

TNF-α, a cytokine with a variety of different functions, has been implicated to play a key role in inflammation and is involved in both chronic inflammation and lipid metabolism. It enhances the production of free fatty acids, induces lipolysis, regulates lipid metabolism associated enzymes and influences cholesterol metabolism. Plasma levels of TNF-α are altered in patients suffering from dyslipidemia and can be regulated by drugs interfering with lipid metabolic pathways [49]. In IBD, TNF-α levels are increased and associated with mucosal inflammation. Treatment with anti-TNF-α-antibodies significantly improved disease activity, which implies a key role for TNF-α in pro-inflammatory signaling [50,51]. Therapeutic application of Infliximab, a chimeric monoclonal antibody which binds to TNF-α with high affinity, was shown to be effective not only in the treatment of Crohn’s disease but also in UC. TNF-α treatment ameliorated disease activity in patients suffering from UC, as shown by the induction of mucosal healing [52], thus pointing out the significant role of TNF-α in IBD pathogenesis.

TNF-α signal transduction has been linked to specialized, liquid ordered areas within cellular membranes, termed lipid rafts [53]. Lipid rafts are dynamic assemblies of lipids, cholesterol and proteins which facilitate signal transduction and regulate membrane function. Cholesterol can be found throughout rafts whereas sphingolipids are located in the outer exoplasmic leaflet, as opposed to phospholipids, that are enriched in the inner cytoplasmic part [54]. In order to form larger platforms, single rafts can cluster together which allows improved signaling by the recruitment of adaptor molecules. Lipid rafts have been implicated in the compartmentalization of membranes, thus generating a heterogenic composition in order to regulate membrane bioactivity [55]. Furthermore, lipid rafts seem to play a role in various pathological conditions, such as viral infections and Alzheimer’s disease and are involved in immune cell signaling [56].

Also in TNF-α mediated signaling, lipid rafts are required for a proper signal transduction. For TNF receptor 1 (TNFR1), it has been shown that binding of TNF-α induces the translocation of the receptor to lipid rafts where a signaling complex is formed by the recruitment of adaptor molecules. Cholesterol depletion disrupted lipid rafts and blocked NF-κB activation, thus proving that these specialized membrane domains are critical for NF-κB signaling [57]. The analysis of TNF receptor CD120a identified its death domain, not only to be necessary for the induction of either apoptosis or NF-κB signaling, but also to be critical for its localization to lipid rafts [58]. The application of exogenous PC to CaCo2 cells changed the compartmentation of TNFR1 and TNFR2 to lipid rafts without interfering with TNF-α receptor binding [59]. Interestingly, this would implicate activation of NF-κB signaling rather than its inhibition. As PC had been shown to display anti-inflammatory properties, it can be speculated that alternative regulatory mechanisms must be involved in the downstream signaling cascade after TNF receptor binding.

TNF-α induced NF-κB activation, as a key event in inflammatory signaling, was analyzed in the human colon carcinoma cell line CaCo2. Exogenous PC application inhibited NF-κB nuclear translocation and activation in a time and dose dependent manner. It can therefore be concluded that TNF-α stimulated NF-κB activation, which is one of the key features in pro-inflammatory signaling pathways, is influenced by PC. The inhibition was most effective when unsaturated fatty acid side chains were present within the PC molecule [34,59], showing the relevance of phospholipid structure in modulating intracellular signaling. Interestingly, already in the 1980s, it could be shown that patients suffering from UC had increased arachidonic acid content in phospholipids found in the inflamed colonic mucosa as compared to healthy controls. Docosahexaenoic acid was also increased, whereas levels of oleic acid and palmitoleic acid were reduced [60]. The observed alterations in the fatty acid composition were attributed to the colonic inflammation and indicated the arachidonic acid metabolism to play a role in UC.

Taken together, experimental outcomes from cell culture assays, the analysis of animal models as well as clinical trials, have demonstrated a positive role of PC in anti-inflammatory signaling by targeting key signaling pathways. PC inhibits phagosomal actin nucleation, interferes with MAP kinase phosphorylation, and reduces expression levels of pro-inflammatory cytokines. Furthermore, TNF receptors are translocated to lipid rafts and activation of NF-κB is diminished by PC treatment, resulting in anti-inflammatory signaling.

## 5. cPLA2α—A Key Regulator in Inflammatory Signaling Downstream of PC?

Decreased levels of PC in mucus obtained from UC patients indicate a defect either in PC synthesis or secretion. Typically, inflammatory processes of UC start in the rectum, concentrate in the colon and with progress of the disease spread towards the coecum. A defect in ileal PC secretion would result in a gradual decrease of mucus PC content from the ileum towards the rectum, consistent with a disturbed barrier function of the mucus. Thus, epithelial cells in the rectum and colon would be at highest risk to be attacked by bacteria which would trigger an inflammatory response. For more information on this issue, the interested reader is referred to a recent review which covers this topic in more detail [61]. As alterations in mucus PC were observed irrespective of the disease activity, the diminished PC content is probably more likely to be a primary cause in UC etiology, rather than a secondary phenomenon [21]. However, an alternative or additional explanation for the diminished PC content, aside from a reduction in PC secretion, would be the increased degradation of PC by phospholipases. As has been previously shown, the arachidonic acid content of PC is increased in the inflamed mucosa of patients suffering from ulcerative colitis [60], which indicates a significance of arachidonic acid metabolism in IBD etiology. In this regard, attention should be paid especially to the cytosolic phospholipase A2α (cPLA2α).

cPLA2α specifically cleaves arachidonic acid from the sn2 position of membrane phospholipids [62], thereby releasing an important precursor of the eicosanoid biosynthesis cascade. Via calcium binding to the N-terminal C2 domain, a physiologically relevant increase of intracellular calcium triggers the translocation of cPLA2α from the cytosol to PC containing membranes, such as the Golgi apparatus, the endoplasmic reticulum or the nuclear envelope [62,63]. Phosphorylation of serine residues, including the phosphorylation at Ser505 by MAP kinases, is thought to stabilize membrane binding of the enzyme and enhance its catalytic activity. The same effect is proposed to be mediated by association of cPLA2α with anionic phospholipids as reviewed by Hirabayashi *et al*. [63]. Co-localization of cPLA2 and MAP kinases has been shown in lipid bodies of human leukemic U937 cells, thereby emphasizing the regulatory function of MAP kinases for cPLA2α activity [64]. Furthermore, the study of ERK1/2, p38 and JNK in a cell culture model revealed that MAP kinases contribute to prostaglandin signaling by regulating the release of arachidonic acid [65].

Indications of the relevance of cPLA2α function in the gastrointestinal system were obtained by mutation of the cPLA2 gene in a mouse model of human familial adenomatous polyposis. The gene mutation decreased the size of intestinal polyps, but had no effect on polyps located in the colon [66]. In a rat model of TNBS induced colon inflammation, the extracellular PLA2 inhibitor carboxymethylcellulose-linked phosphatidylethanolamine (CMPE) improved the disease status and reduced the mortality rate [67]. Although this effect is likely to be mediated via the inhibition of secretory rather than cytoplasmic PLA2, it outlines the importance of cPLA activity in intestinal inflammation in general.

In human, an adult male with inherited cPLA2α deficiency displayed selective ulceration in ileum and jejunum together with gastrointestinal blood loss and dysfunction in eicosanoid production as well as significantly reduced arachidonic acid levels in platelet activation [68]. Thus, functional impairment or complete loss of cPLA2α function resulted in damage to the gastrointestinal tract. This might suggest a specific role for cPLA2α in maintaining the homeostasis and integrity in this part of the digestive system. Although the increased degradation of PC by cPLA2α overexpression may mediate pro-inflammatory properties, the impaired function of cPLA2α equally causes malfunctions, thus hinting at a pro-inflammatory as well as anti-inflammatory role of phospholipases [69].

cPLA2α acts through the hydrolysis of membrane phospholipids that release free arachidonic acid which are further used for downstream metabolite production. In a CaCo2 cell model, LPC was found to increase calcium levels and decrease the transepithelial electrical resistance (TEER) due to altered tight-junction permeability [70]. An impaired TEER facilitates bacterial translocation and might therefore contribute to the development and maintenance of inflammatory processes. However, cell culture assays also showed a reduced NF-κB activation by LPC treatment [34], indicating a complex role for LPC within inflammatory signaling.

Beside the impact of LPC, arachidonic acid is likely to be a key factor in mediating the effects of cPLA2α activity. Arachidonic acid can be processed either via the cyclooxygenase (COX) pathway or the lipoxygenase (LOX) pathway, thus leading to the production of lipid mediators that might be involved in the pathogenesis of IBD. It is likely, that selective blocking of one pathway redirects arachidonic acid metabolism to the alternative pathway, which might be an explanation for the induction of gastrointestinal damage by NSAID meditated COX inhibition. Simultaneous blocking of both pathways showed good anti-inflammatory effects and reduced gastrointestinal toxicity [71].

Synthesis of arachidonic acid is the rate limiting step in prostaglandin production [62] via the COX pathway. Prostaglandins, especially prostaglandin E2 (PGE2), have been shown to exhibit gastroprotective mechanisms: Intragastric injection of rats with PGE2 resulted in enhanced phospholipid levels in the gastric mucosa, an effect which was most prominent for PC [4]. Furthermore, prostaglandins increased the gastric surface hydrophobicity and stimulated lipid biosynthesis (reviewed by Lichtenberger [5]). The parenteral administration of NSAIDs in healthy rat caused decreased hydrophobicity of the gastric and duodenal mucosa. This effect could be linked to defects in prostaglandin generation in addition to which the involvement of other factors is likely [7]. Prostaglandins have not only been implicated in gastrointestinal inflammation but they also seem to play a role in colon tumor development. Colon cancer cell lines specifically showed increased levels of PGE2 and COX2 when cultured in a hypotonic environment as opposed to cell lines derived from non-tumor intestinal epithelial cell lines [65]. An analysis of eight colorectal cancer cell lines and 65 colorectal cancers showed an overexpression of cPLA2 in about 50% of the cases which correlated with COX2 expression in a positive manner [72].

Taken together, experimental findings show the involvement of cPLA2α in the regulation of various pathological conditions, its effect resulting from the degradation of membrane PC. Pro-inflammatory, as well as anti-inflammatory properties of cPLA2α, indicate the need for a tight regulation of its function, which has to be taken into account when addressed as a therapeutic target. For this reason, not complete inhibition but rather a reduction in activity to basal levels might be the best method of choice [69].

## 6. Perspectives

The development of ulcerative colitis, an autoaggressive, chronic inflammatory bowel disease, can be attributed to a misbalanced inflammatory response to luminal pathogens within the gastrointestinal system. Although the underlying signaling mechanisms remain incompletely understood, it has been shown that PC plays an important role in establishing a protective mucosal barrier as an essential part of the intestinal mucus. Furthermore, PC might be integrated into phospholipid membranes of enterocytes, thereby altering downstream signaling cascades. The exogenous application of PC in patients suffering from ulcerative colitis, ameliorated clinical parameters and acted in an anti-inflammatory manner. Beneficial effects of PC treatments were also evident in animal models of (chronic) inflammatory diseases.

PC increases hydrophobicity on the luminal surface of the GI-tract, inhibits actin nucleation, TNF-α mediated NF-κB nuclear translocation, MAP kinase activation and pro-inflammatory cytokine expression. It might translocate TNF receptors to lipid rafts, influencing eicosanoid production via interaction with cPLA2α. Light has been shed on a variety of possible PC dependent pathways, suggesting that it is required to “fine tune” the complex signaling network which regulates inflammatory processes. Although many open questions remain to be solved, the presently available data strongly encourage further investigations of PC involvement in inflammation. It is tempting to speculate that the modulation of PC dependent signaling might in future be highly relevant in establishing new therapies for IBD treatment, especially ulcerative colitis.

## References

[1] Best CH Hershey JM Huntsman ME The effect of lecithine on fat deposition in the liver of the normal rat J. Physiol 1932 75 56 66 16994301 10.1113/jphysiol.1932.sp002875 PMC1394511

[2] Eros G Ibrahim S Siebert N Boros M Vollmar B Oral phosphatidylcholine pretreatment alleviates the signs of experimental rheumatoid arthritis Arthritis Res. Ther 2009 11 R43 19296835 10.1186/ar2651 PMC2688190

[3] Eros G Varga G Varadi R Czobel M Kaszaki J Ghyczy M Boros M Anti-inflammatory action of a phosphatidylcholine, phosphatidylethanolamine and *N*-acylphosphatidylethanolamine-enriched diet in carrageenan-induced pleurisy Eur. Surg. Res 2009 42 40 48 18987473 10.1159/000167856

[4] Lichtenberger LM Graziani LA Dial EJ Butler BD Hills BA Role of surface-active phospholipids in gastric cytoprotection Science 1983 219 1327 1329 6828859 10.1126/science.6828859

[5] Lichtenberger LM The hydrophobic barrier properties of gastrointestinal mucus Annu. Rev. Physiol 1995 57 565 583 7778878 10.1146/annurev.ph.57.030195.003025

[6] Zhou Y Dial EJ Doyen R Lichtenberger LM Effect of indomethacin on bile acid-phospholipid interactions: Implication for small intestinal injury induced by nonsteroidal anti-inflammatory drugs Am. J. Physiol. Gastrointest. Liver Physiol 2010 298 G722 G731 20203063 10.1152/ajpgi.00387.2009 PMC2867422

[7] Lugea A Antolin M Mourelle M Guarner F Malagelada JR Deranged hydrophobic barrier of the rat gastroduodenal mucosa after parenteral nonsteroidal anti-inflammatory drugs Gastroenterology 1997 112 1931 1939 9178685 10.1053/gast.1997.v112.pm9178685

[8] Lichtenberger LM Romero JJ Dial EJ Gastrointestinal safety and therapeutic efficacy of parenterally administered phosphatidylcholine-associated indomethacin in rodent model systems Br. J. Pharmacol 2009 157 252 257 19366347 10.1111/j.1476-5381.2009.00159.x PMC2697803

[9] Lanza FL Marathi UK Anand BS Lichtenberger LM Clinical trial: Comparison of ibuprofen-phosphatidylcholine and ibuprofen on the gastrointestinal safety and analgesic efficacy in osteoarthritic patients Aliment Pharmacol. Ther 2008 28 431 442 18549459 10.1111/j.1365-2036.2008.03765.x PMC3353548

[10] Cryer B Bhatt DL Lanza FL Dong J-F Lichtenberger LM Marathi UK Reduction of gastroduodenal ulceration with aspirin-phosphatidylcholine complex *versus* aspirin—potential importance of local mucosal injury Gastroenterology 2010 138 S497

[11] Podolsky DK Inflammatory bowel disease N. Engl. J. Med 2002 347 417 429 12167685 10.1056/NEJMra020831

[12] Sartor RB Therapeutic manipulation of the enteric microflora in inflammatory bowel diseases: Antibiotics, probiotics, and prebiotics Gastroenterology 2004 126 1620 1633 15168372 10.1053/j.gastro.2004.03.024

[13] Backhed F Ley RE Sonnenburg JL Peterson DA Gordon JI Host-bacterial mutualism in the human intestine Science 2005 307 1915 1920 15790844 10.1126/science.1104816

[14] Rutgeerts P Goboes K Peeters M Hiele M Penninckx F Aerts R Kerremans R Vantrappen G Effect of faecal stream diversion on recurrence of Crohn's disease in the neoterminal ileum Lancet 1991 338 771 774 1681159 10.1016/0140-6736(91)90663-a

[15] Duchmann R May E Heike M Knolle P Neurath M Meyer zum Buschenfelde KH T cell specificity and cross reactivity towards enterobacteria, bacteroides, bifidobacterium, and antigens from resident intestinal flora in humans Gut 1999 44 812 818 10323882 10.1136/gut.44.6.812 PMC1727529

[16] Swidsinski A Ladhoff A Pernthaler A Swidsinski S Loening-Baucke V Ortner M Weber J Hoffmann U Schreiber S Dietel M Lochs H Mucosal flora in inflammatory bowel disease Gastroenterology 2002 122 44 54 11781279 10.1053/gast.2002.30294

[17] Wen Z Fiocchi C Inflammatory bowel disease: Autoimmune or immune-mediated pathogenesis? Clin. Dev. Immunol 2004 11 195 204 15559364 10.1080/17402520400004201 PMC2486322

[18] Pullan RD Thomas GA Rhodes M Newcombe RG Williams GT Allen A Rhodes J Thickness of adherent mucus gel on colonic mucosa in humans and its relevance to colitis Gut 1994 35 353 359 8150346 10.1136/gut.35.3.353 PMC1374589

[19] Einerhand AW Renes IB Makkink MK van der Sluis M Buller HA Dekker J Role of mucins in inflammatory bowel disease: Important lessons from experimental models Eur. J. Gastroenterol. Hepatol 2002 14 757 765 12169985 10.1097/00042737-200207000-00008

[20] Ehehalt R Wagenblast J Erben G Lehmann WD Hinz U Merle U Stremmel W Phosphatidylcholine and lysophosphatidylcholine in intestinal mucus of ulcerative colitis patients. A quantitative approach by nanoElectrospray-tandem mass spectrometry Scand. J. Gastroenterol 2004 39 737 742 15513358 10.1080/00365520410006233

[21] Braun A Treede I Gotthardt D Tietje A Zahn A Ruhwald R Schoenfeld U Welsch T Kienle P Erben G Lehmann WD Fuellekrug J Stremmel W Ehehalt R Alterations of phospholipid concentration and species composition of the intestinal mucus barrier in ulcerative colitis: A clue to pathogenesis Inflamm. Bowel. Dis 2009 15 1705 1720 19504612 10.1002/ibd.20993

[22] Longman RJ Poulsom R Corfield AP Warren BF Wright NA Thomas MG Alterations in the composition of the supramucosal defense barrier in relation to disease severity of ulcerative colitis J. Histochem. Cytochem 2006 54 1335 1348 16924127 10.1369/jhc.5A6904.2006 PMC3958115

[23] Wehkamp J Schmid M Stange EF Defensins and other antimicrobial peptides in inflammatory bowel disease Curr. Opin. Gastroenterol 2007 23 370 378 17545771 10.1097/MOG.0b013e328136c580

[24] Schulzke JD Ploeger S Amasheh M Fromm A Zeissig S Troeger H Richter J Bojarski C Schumann M Fromm M Epithelial tight junctions in intestinal inflammation Ann. N.Y. Acad. Sci 2009 1165 294 300 19538319 10.1111/j.1749-6632.2009.04062.x

[25] Langmann T Moehle C Mauerer R Scharl M Liebisch G Zahn A Stremmel W Schmitz G Loss of detoxification in inflammatory bowel disease: Dysregulation of pregnane X receptor target genes Gastroenterology 2004 127 26 40 15236169 10.1053/j.gastro.2004.04.019

[26] Macfarlane GT Blackett KL Nakayama T Steed H Macfarlane S The gut microbiota in inflammatory bowel disease Curr. Pharm. Des 2009 15 1528 1536 19442170 10.2174/138161209788168146

[27] Fabia R Ar'Rajab A Willen R Andersson R Ahren B Larsson K Bengmark S Effects of phosphatidylcholine and phosphatidylinositol on acetic-acid-induced colitis in the rat Digestion 1992 53 35 44 1289171 10.1159/000200969

[28] Fabia R Ar'Rajab A Willen R Andersson R Bengmark S Effect of putative phospholipase A2 inhibitors on acetic acid-induced acute colitis in the rat Br. J. Surg 1993 80 1199 1204 8402133 10.1002/bjs.1800800947

[29] Tatsumi Y Lichtenberger LM Molecular association of trinitrobenzenesulfonic acid and surface phospholipids in the development of colitis in rats Gastroenterology 1996 110 780 789 8608888 10.1053/gast.1996.v110.pm8608888

[30] Mourelle M Guarner F Malagelada JR Polyunsaturated phosphatidylcholine prevents stricture formation in a rat model of colitis Gastroenterology 1996 110 1093 1097 8612998 10.1053/gast.1996.v110.pm8612998

[31] Barrios JM Lichtenberger LM Role of biliary phosphatidylcholine in bile acid protection and NSAID injury of the ileal mucosa in rats Gastroenterology 2000 118 1179 1186 10833493 10.1016/s0016-5085(00)70371-4

[32] Lugea A Salas A Casalot J Guarner F Malagelada JR Surface hydrophobicity of the rat colonic mucosa is a defensive barrier against macromolecules and toxins Gut 2000 46 515 521 10716681 10.1136/gut.46.4.515 PMC1727902

[33] Sturm A Zeeh J Sudermann T Rath H Gerken G Dignass AU Lisofylline and lysophospholipids ameliorate experimental colitis in rats Digestion 2002 66 23 29 12379812 10.1159/000064418

[34] Treede I Braun A Sparla R Kuhnel M Giese T Turner JR Anes E Kulaksiz H Fullekrug J Stremmel W Griffiths G Ehehalt R Anti-inflammatory effects of phosphatidylcholine J. Biol. Chem 2007 282 27155 27164 17636253 10.1074/jbc.M704408200 PMC2693065

[35] Anes E Kuhnel MP Bos E Moniz-Pereira J Habermann A Griffiths G Selected lipids activate phagosome actin assembly and maturation resulting in killing of pathogenic mycobacteria Nat. Cell Biol 2003 5 793 802 12942085 10.1038/ncb1036

[36] Gutierrez MG Gonzalez AP Anes E Griffiths G Role of lipids in killing mycobacteria by macrophages: Evidence for NF-kappaB-dependent and -independent killing induced by different lipids Cell Microbiol 2009 11 406 420 19016780 10.1111/j.1462-5822.2008.01263.x

[37] Stremmel W Merle U Zahn A Autschbach F Hinz U Ehehalt R Retarded release phosphatidylcholine benefits patients with chronic active ulcerative colitis Gut 2005 54 966 971 15951544 10.1136/gut.2004.052316 PMC1774598

[38] Tromm A Griga T May B Oral mesalazine for the treatment of Crohn's disease: Clinical efficacy with respect to pharmacokinetic properties Hepatogastroenterology 1999 46 3124 3135 10626173

[39] Stremmel W Braun A Hanemann A Ehehalt R Autschbach F Karner M Delayed release phosphatidylcholine in chronic-active ulcerative colitis: A randomized, double-blinded, dose finding study J. Clin. Gastroenterol 2010 44 e101 107 20048683 10.1097/MCG.0b013e3181c29860

[40] Stremmel W Ehehalt R Autschbach F Karner M Phosphatidylcholine for steroid-refractory chronic ulcerative colitis: A randomized trial Ann. Int. Med 2007 147 603 610 17975182 10.7326/0003-4819-147-9-200711060-00004

[41] Parlesak A Schaeckeler S Moser L Bode C Conjugated primary bile salts reduce permeability of endotoxin through intestinal epithelial cells and synergize with phosphatidylcholine in suppression of inflammatory cytokine production Crit. Care Med 2007 35 2367 2374 17944028 10.1097/01.ccm.0000284586.84952.fb

[42] Dial EJ Tran DM Romero JJ Zayat M Lichtenberger LM A direct role for secretory phospholipase A2 and lyso-phosphatidylcholine in the mediation of lipopolysaccharide-induced gastric injury Shock 2010 33 634 638 19940811 10.1097/SHK.0b013e3181cb9266 PMC2875268

[43] Hills BA Surface-active phospholipid: A Pandora's box of clinical applications. Part II. Barrier and lubricating properties Int. Med. J 2002 32 242 251 10.1046/j.1445-5994.2002.00201.x 12036223

[44] Braun A Schoenfeld U Welsch T Kadmon M Funke B Autschbach F Grunze M Stremmel W Kienle P Ehehalt R The surface hydrophobicity of the colonic mucosa is reduced in ulcerative colitis J. Crohn's Colitis 2010 4 S13 S120

[45] Hurley JH Tsujishita Y Pearson MA Floundering about at cell membranes: A structural view of phospholipid signaling Curr. Opin. Struct. Biol 2000 10 737 743 11114512 10.1016/s0959-440x(00)00144-5

[46] Alpy F Tomasetto C Give lipids a START: The StAR-related lipid transfer (START) domain in mammals J. Cell Sci 2005 118 2791 2801 15976441 10.1242/jcs.02485

[47] Medina-Gomez G Gray SL Yetukuri L Shimomura K Virtue S Campbell M Curtis RK Jimenez-Linan M Blount M Yeo GS Lopez M Seppanen-Laakso T Ashcroft FM Oresic M Vidal-Puig A PPAR gamma 2 prevents lipotoxicity by controlling adipose tissue expandability and peripheral lipid metabolism PLoS Genet 2007 3 e64 17465682 10.1371/journal.pgen.0030064 PMC1857730

[48] Chakravarthy MV Lodhi IJ Yin L Malapaka RR Xu HE Turk J Semenkovich CF Identification of a physiologically relevant endogenous ligand for PPARalpha in liver Cell 2009 138 476 488 19646743 10.1016/j.cell.2009.05.036 PMC2725194

[49] Chen X Xun K Chen L Wang Y TNF-alpha, a potent lipid metabolism regulator Cell Biochem. Funct 2009 27 407 416 19757404 10.1002/cbf.1596

[50] Targan SR Hanauer SB van Deventer SJ Mayer L Present DH Braakman T DeWoody KL Schaible TF Rutgeerts PJ A short-term study of chimeric monoclonal antibody cA2 to tumor necrosis factor alpha for Crohn's disease. Crohn's Disease cA2 Study Group N. Engl. J. Med 1997 337 1029 1035 9321530 10.1056/NEJM199710093371502

[51] van Deventer SJ Tumour necrosis factor and Crohn's disease Gut 1997 40 443 448 9176068 10.1136/gut.40.4.443 PMC1027115

[52] Rutgeerts P Sandborn WJ Feagan BG Reinisch W Olson A Johanns J Travers S Rachmilewitz D Hanauer SB Lichtenstein GR de Villiers WJ Present D Sands BE Colombel JF Infliximab for induction and maintenance therapy for ulcerative colitis N. Engl. J. Med 2005 353 2462 2476 16339095 10.1056/NEJMoa050516

[53] Hueber AO Role of membrane microdomain rafts in TNFR-mediated signal transduction Cell Death Differ 2003 10 7 9 12655288 10.1038/sj.cdd.4401155

[54] Simons K Ehehalt R Cholesterol, lipid rafts, and disease J. Clin. Invest 2002 110 597 603 12208858 10.1172/JCI16390 PMC151114

[55] Lingwood D Simons K Lipid rafts as a membrane-organizing principle Science 2010 327 46 50 20044567 10.1126/science.1174621

[56] Triantafilou M Morath S Mackie A Hartung T Triantafilou K Lateral diffusion of Toll-like receptors reveals that they are transiently confined within lipid rafts on the plasma membrane J. Cell Sci 2004 117 4007 4014 15286178 10.1242/jcs.01270

[57] Legler DF Micheau O Doucey MA Tschopp J Bron C Recruitment of TNF receptor 1 to lipid rafts is essential for TNFalpha-mediated NF-kappaB activation Immunity 2003 18 655 664 12753742 10.1016/s1074-7613(03)00092-x

[58] Cottin V Doan JE Riches DW Restricted localization of the TNF receptor CD120a to lipid rafts: A novel role for the death domain J. Immunol 2002 168 4095 4102 11937569 10.4049/jimmunol.168.8.4095

[59] Treede I Braun A Jeliaskova P Giese T Fullekrug J Griffiths G Stremmel W Ehehalt R TNF-alpha-induced up-regulation of pro-inflammatory cytokines is reduced by phosphatidylcholine in intestinal epithelial cells BMC Gastroenterol 2009 9 53 19594939 10.1186/1471-230X-9-53 PMC2714528

[60] Nishida T Miwa H Shigematsu A Yamamoto M Iida M Fujishima M Increased arachidonic acid composition of phospholipids in colonic mucosa from patients with active ulcerative colitis Gut 1987 28 1002 1007 3117625 10.1136/gut.28.8.1002 PMC1433134

[61] Ehehalt R Braun A Karner M Fullekrug J Stremmel W Phosphatidylcholine as a constituent in the colonic mucosal barrier--physiological and clinical relevance Biochim. Biophys. Acta 2010 1801 983 993 20595010 10.1016/j.bbalip.2010.05.014

[62] Clark JD Lin LL Kriz RW Ramesha CS Sultzman LA Lin AY Milona N Knopf JL A novel arachidonic acid-selective cytosolic PLA2 contains a Ca^2+^-dependent translocation domain with homology to PKC and GAP Cell 1991 65 1043 1051 1904318 10.1016/0092-8674(91)90556-e

[63] Hirabayashi T Murayama T Shimizu T Regulatory mechanism and physiological role of cytosolic phospholipase A2 Biol. Pharm. Bull 2004 27 1168 1173 15305015 10.1248/bpb.27.1168

[64] Yu W Bozza PT Tzizik DM Gray JP Cassara J Dvorak AM Weller PF Co-compartmentalization of MAP kinases and cytosolic phospholipase A2 at cytoplasmic arachidonate-rich lipid bodies Am. J. Pathol 1998 152 759 769 9502418 PMC1858398

[65] Gentile LB Piva B Capizzani BC Furlaneto LG Moreira LS Zamith-Miranda D Diaz BL Hypertonic environment elicits cyclooxygenase-2-driven prostaglandin E2 generation by colon cancer cells: Role of cytosolic phospholipase A2-alpha and kinase signaling pathways Prostagl. Leukot Essent Fat. Ac 2010 82 131 139 10.1016/j.plefa.2009.11.005 20004562

[66] Takaku K Sonoshita M Sasaki N Uozumi N Doi Y Shimizu T Taketo MM Suppression of intestinal polyposis in Apc(delta 716) knockout mice by an additional mutation in the cytosolic phospholipase A(2) gene J. Biol. Chem 2000 275 34013 34016 10969066 10.1074/jbc.C000585200

[67] Krimsky M Yedgar S Aptekar L Schwob O Goshen G Gruzman A Sasson S Ligumsky M Amelioration of TNBS-induced colon inflammation in rats by phospholipase A2 inhibitor Am. J. Physiol. Gastrointest. Liver Physiol 2003 285 G586 592 12724134 10.1152/ajpgi.00463.2002

[68] Adler DH Cogan JD Phillips JA 3rd Schnetz-Boutaud N Milne GL Iverson T Stein JA Brenner DA Morrow JD Boutaud O Oates JA Inherited human cPLA(2alpha) deficiency is associated with impaired eicosanoid biosynthesis, small intestinal ulceration, and platelet dysfunction J. Clin. Invest 2008 118 2121 2131 18451993 10.1172/JCI30473 PMC2350426

[69] Yedgar S Cohen Y Shoseyov D Control of phospholipase A2 activities for the treatment of inflammatory conditions Biochim. Biophys. Acta 2006 1761 1373 1382 16978919 10.1016/j.bbalip.2006.08.003

[70] Sawai T Lampman R Hua Y Segura B Drongowski RA Coran AG Harmon CM Lysophosphatidylcholine alters enterocyte monolayer permeability via a protein kinase C/Ca^2+^ mechanism Pediatr. Surg. Int 2002 18 591 594 12471472 10.1007/s00383-002-0860-x

[71] Tries S Neupert W Laufer S The mechanism of action of the new antiinflammatory compound ML3000: Inhibition of 5-LOX and COX-1/2 Inflamm. Res 2002 51 135 143 12005204 10.1007/pl00000285

[72] Panel V Boelle PY Ayala-Sanmartin J Jouniaux AM Hamelin R Masliah J Trugnan G Flejou JF Wendum D Cytoplasmic phospholipase A2 expression in human colon adenocarcinoma is correlated with cyclooxygenase-2 expression and contributes to prostaglandin E2 production Cancer Lett 2006 243 255 263 16458424 10.1016/j.canlet.2005.11.045

